# Detection of Fusion Genes Using a Targeted RNA Sequencing Panel in Gastrointestinal and Rare Cancers

**DOI:** 10.1155/2020/4659062

**Published:** 2020-01-22

**Authors:** Su Jin Lee, Jung Yong Hong, Kyung Kim, Kyoung-Mee Kim, So Young Kang, Taeyang Lee, Seung Tae Kim, Se Hoon Park, Young Suk Park, Ho Yeong Lim, Won Ki Kang, Jeeyun Lee, Joon Oh Park

**Affiliations:** ^1^Division of Hematology-Oncology, Department of Medicine, Sungkyunkwan University School of Medicine, Seoul, Republic of Korea; ^2^Division of Hematology-Oncology, Department of Internal Medicine, Ewha Womans University College of Medicine, Seoul, Republic of Korea; ^3^Department of Pathology, Samsung Medical Center, Sungkyunkwan University School of Medicine, Seoul, Republic of Korea

## Abstract

Successful identification and targeting of oncogenic gene fusion is a major breakthrough in cancer treatment. Here, we investigate the therapeutic implications and feasibility of using a targeted RNA sequencing panel to identify fusion genes in gastrointestinal and rare cancers. From February through December 2017, patients with gastrointestinal, hepatobiliary, gynecologic, sarcoma, or rare cancers were recruited for a clinical sequencing project at Samsung Medical Center (NCT #02593578). The median age of the patients was 58 years (range, 31–81 years), and the male-to-female ratio was 1.3 : 1. A total of 118 patients passed the quality control process for a next-generation sequencing- (NGS-) based targeted sequencing assay. The NGS-based targeted sequencing assay was performed to detect gene fusions in 36–53 cancer-implicated genes. The following cancer types were included in this study: 28 colorectal cancers, 27 biliary tract cancers, 25 gastric cancers, 18 soft tissue sarcomas, 9 pancreatic cancers, 6 ovarian cancers, and 9 other rare cancers. Strong fusion was detected in 25 samples (21.2%). We found that 5.9% (7/118) of patients had known targetable fusion genes involving *NTRK1* (*n*=3), *FGFR* (*n*=3), and *RET* (*n*=1), and 10.2% (12/118) of patients had potentially targetable fusion genes involving *RAF1* (*n*=4), *BRAF* (*n*=2), *ALK* (*n*=2), *ROS1* (*n*=1), *EGFR* (*n*=1), and *CLDN18* (*n*=2). Thus, we successfully identified a substantial proportion of patients harboring fusion genes by RNA panel sequencing of gastrointestinal/rare cancers. Targetable and potentially targetable involved fusion genes were *NTRK1*, *RET*, *FGFR3*, *FGFR2*, *BRAF*, *RAF1*, *ALK*, *ROS1*, and *CLDN18*. Detection of fusion genes by RNA panel sequencing may be beneficial in refractory patients with gastrointestinal/rare cancers.

## 1. Introduction

Successful identification and targeting of oncogenic gene fusion has been one of the major breakthroughs in cancer treatment in recent decades [1–3]. Generally, the prevalence of gene fusion is lower than that of oncogenic somatic mutations in solid cancers. However, techniques for fusion detection revealed that up to 17% of solid cancers harbored at least one gene fusion [3]. Oncogenic gene fusions frequently involve tyrosine kinases and can cause constitutive activation of tyrosine kinases, augmentation of downstream survival signal, and progression of cancer. Remarkable success has been achieved by targeting oncogenic gene fusions including diverse tyrosine kinase inhibitors against fusions involving *ALK*, *ROS1*, *RET*, *FGFR1/2/3*, and *NTRK1/2/3* in non-small-cell lung cancer and across a wide spectrum of cancer types [4–7].

Gene fusions can be formed by various types of chromosomal breakage and rejoining events, including translocations, inversions, deletions, and duplications [1–3]. Common methods for detecting fusions in the clinic include break-apart fluorescence *in situ* hybridization (FISH), reverse transcription polymerase chain reaction (RT-PCR), and next-generation sequencing (NGS) [1–3]. The first two methods show high sensitivity for fusion detection but typically test for a single fusion gene and cannot detect novel fusion partners or complex structural rearrangements; they are also less sensitive for detecting intrachromosomal fusion genes. Whole genome sequencing (WGS) and whole transcriptome sequencing (RNA sequencing) are two major NGS technologies used for fusion gene detection [3]. WGS provides the most comprehensive characterization of genomic alterations in cancer genomes. However, WGS requires greater sequencing effort and intensive analysis. Additionally, the significance of fusion genes discovered by WGS must be re-evaluated to determine whether fusion RNA transcripts are produced. RNA sequencing only sequences regions of the genome that are transcribed and spliced into mature mRNA. Thus, RNA sequencing is less costly and time-consuming and can detect multiple alternative fusion variants. Most recent studies that discovered novel gene fusions have used RNA sequencing platforms. Here, we investigated the therapeutic implications and feasibility of using a targeted RNA sequencing panel to identify fusion genes in gastrointestinal and rare cancers.

## 2. Materials and Methods

### 2.1. Patients

From February through December 2017, 122 patients with gastrointestinal, hepatobiliary, gynecologic, sarcoma, or other rare cancers participated in the clinical sequencing project for evaluation with the NGS-based targeted sequencing assay (Archer® FusionPlex, ArcherDx, Boulder, CO, USA) at Samsung Medical Center (NCT #02593578). In brief, patients with metastatic solid cancers in whom standard chemotherapy had failed or rare cancers who were not treated by standard chemotherapy were enrolled in the study. All patients signed informed consent forms to participate in the study, and the study protocol was approved by the institutional review board of Samsung Medical Center.

### 2.2. Targeted RNA Panel Sequencing

We used the NGS-based targeted sequencing assay to detect gene fusion in 36–53 cancer-implicated genes (Archer® FusionPlex). Anchored multiplex PCR was performed for targeted RNA sequencing using the ArcherDx fusion assay (Archer® FusionPlex Comprehensive Thyroid & Lung (CTL) kit or Solid Tumor kit). Thirty-six genes in the CTL kit and 53 genes in the solid tumor kit are listed in Supplementary Tables 1 and 2. Formalin-fixed, paraffin-embedded tumor samples were microdissected to enrich the sample to ≥20% tumor nuclei, and total nucleic acid was extracted from the FFPE patient sample using AllPrep DNA/RNA FFPE kit according to the manufacturer's recommended protocol (Qiagen, Valencia, CA). First‐ and second‐strand complementary DNA (cDNA) synthesis was performed. Unidirectional gene-specific primers were used to enrich target regions, followed by NGS with the Illumina MiSeq platform (San Diego, CA, USA). The produced libraries were analyzed for the presence of relevant fusions. Reads matching a database of known fusions and other oncogenic isoforms (Quiver database, ArcherDx) as well as novel isoforms or fusions with high reads (>10% of total reads) and high confidence after bioinformatic filtering were analyzed. Samples with <4,000 unique RNA reads were reported as indeterminate and excluded from analysis. All analyzed fusions were in-frame and predicted to have preserved kinase domains. Fusions among the >11,000 fusions known to be present in normal tissues were excluded [8]. The clinical literature was reviewed to determine the therapeutic implications of the identified fusions.

### 2.3. Fish

To validate the NTRK1 gene rearrangements by FISH, we used the ZytoLight SPEC NTRK1 Dual Color Break Apart Probe (ZytoVision, Bremerhaven, Germany) according to the operating instructions [9]. Using appropriate filter sets, the interphases of normal cells or cells without a translocation involving the 1q23.1 band show two green/orange fusion signals. A 1q23.1 locus affected by a translocation is indicated by one separate green signal and one separate orange signal. A threshold of 15% nuclei positive for break apart signals was used to establish the cutoff for positive FISH results.

## 3. Results

### 3.1. Patient Characteristics

Patient characteristics are shown in Table 1. Sixty-eight patients (55.7%) were male, and the median age of the patients was 58 years (range, 31–81 years). Patients included in this study had various types of cancer: 28 patients with colorectal cancer (CRC), 27 with biliary tract cancer (BTC), 25 with gastric cancer (GC), 18 with soft tissue sarcoma (STS), 9 with pancreatic cancer, 6 with ovarian cancer, and 9 with other rare cancers. Fifty-eight patients (45.1%) showed metastatic disease at initial presentation. The most common metastatic sites were as follows: liver (31.4%), peritoneal seeding (27.3%), lung (22.3%), lymph node (22.3%), bone (10.7%), ovary (7.4%), and pleura (5.8%).

### 3.2. Detection of Fusion Genes

Among the 122 cases, 118 cases (96.7%) passed the quality control process for the NGS-based targeted sequencing assay. Overall, we observed 28 fusion events in 25 cases (21.2%, 25/118), and 3 cases showed 2 types of fusion transcripts. Cancer types in which a fusion was detected were CRC (*n*=7), STS (*n*=6), BTC (*n*=5), GC (*n*=5), melanoma (*n*=1), and pancreatic cancer (*n*=1). The detection rates of fusion genes were 25.0% in CRC (7/28), 33.3% in STS (6/18), 18.5% in BTC (5/27), 20.0% in GC (5/25), 100% in melanoma (1/1), and 11.1% in pancreatic cancer (1/9). No fusion genes were detected in gynecologic cancers (0/9). Patient numbers, detailed descriptions of the cancer types, histology, and identified fusion genes are shown in Table 2.

**Figure 1:**
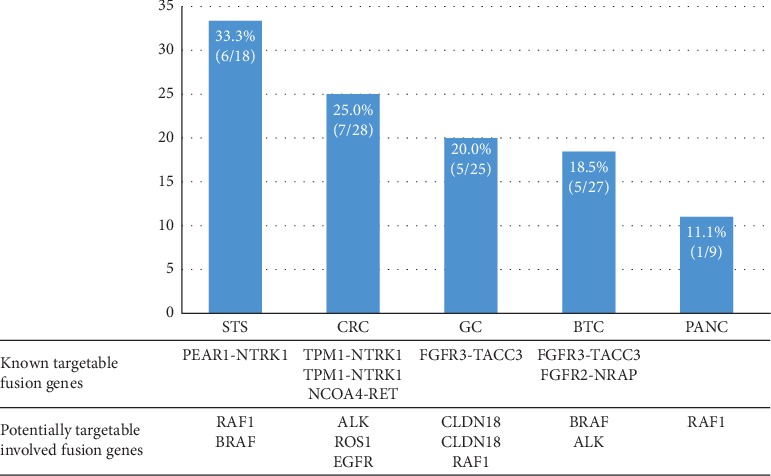
Detection rate of fusion genes and targetable fusion genes according to cancer types.

Notably, known therapeutically targetable fusions were identified in 7 cases (5.9%): two CRCs with *TPM1-NTRK1* fusion, one STS with *PEAR1-NTRK1* fusion, one CRC with *NCOA4-RET* fusion, one GC and one BTC with *FGFR3-TACC3* fusion, and one BTC with *FGFR2-NRAP* fusion. Additionally, potentially targetable fusions were found in 12 patients (10.2%). *RAF1* fusion was detected in 4 cases (GC, melanoma, STS, and pancreatic cancer), *BRAF* fusion in 2 cases (BTC and STS), *ALK* fusion in 2 cases (CRC and BTC), *ROS1* fusion in 1 case (CRC), and *EGFR* fusion in 1 case (CRC) with diverse counterparts. *CLDN18-ARHGAP26* fusion was detected in two cases of GC in this study (8%, 2/25), which was recently reported and investigated in signet-ring GC and diffuse-type GC [10, 11]. The detection rate of fusion genes, targetable fusion genes, and potentially targetable-involved fusion genes according to the cancer types are illustrated in Figure 1.

### 3.3. STS with PEAR1-NTRK1 Fusion

**Figure 2:**
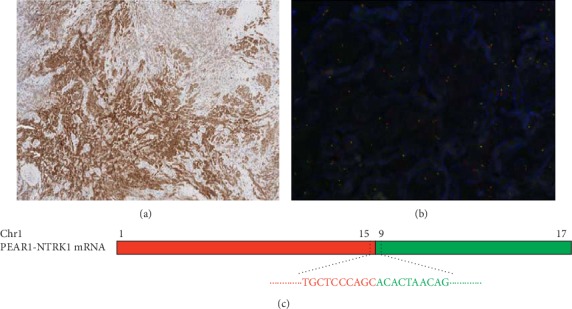
(a) Positive immunohistochemical staining for TRK. (b) Positive FISH analysis for *NTRK1* fusion. (c) *PEAR-NTRK1* fusion confirmed by NGS.

**Figure 3:**
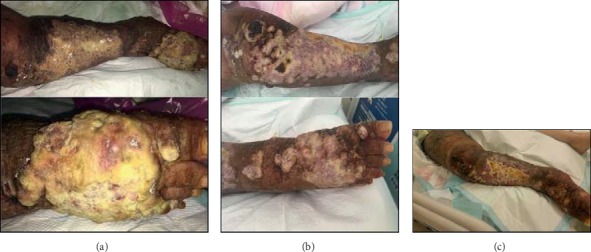
(a) Right lower leg lesion before treatment of TRK inhibitor. (b and c) Right lower leg lesion after 1 cycle of TRK inhibitor.

A novel *PEAR1-NTRK1* fusion was detected in a 78-year-old female patient with angiosarcoma. She initially presented with diffuse infiltrative skin lesion in the right lower leg in December 2016 and had been administered a palliative paclitaxel, pazopanib, and ifosfamide-based combination. However, the patient showed a refractory disease course. She also underwent palliative radiotherapy to the right lower leg for wound management. Based on the *PEAR1-NTRK1* fusion detection in this study, we performed immunohistochemical staining and FISH. The patient showed strong positivity for TRK immunohistochemical staining (Figure 2(a)), and *NTRK1* fusion was confirmed by FISH analysis (Figure 2(b)). We enrolled this patient in a phase I basket trial for treatment with a TRK inhibitor. The tumor was confirmed by NGS to harbor a novel *PEAR1-NTRK1* fusion with the 5′ end of *NTRK1*, including the kinase domain, starting at exon 9 fused to exon 15 of *PEAR1* (Figure 2(c)). The primary lesion in the right lower leg responded well to the TRK inhibitor (Figure 3), but she died of sepsis due to wound infection during the second cycle of treatment.

### 3.4. Melanoma with MAPRE2-RAF1 Fusion

**Figure 4:**
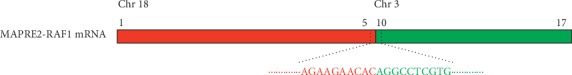
*MAPRE2-RAF1* fusion confirmed by NGS.

A 35-year-old female patient with melanoma showed *MAPRE2-RAF1* fusion. She had previously been treated by surgical resection of the primary melanoma in the lower leg followed by administration of adjuvant interferon therapy. She showed lymph node, lung, liver, bone, and brain metastases and was subsequently treated as follows: pembrolizumab, ipilimumab, dacarbazine-based combination therapy, gamma knife surgery, craniotomy, and tumor removal from the brain. After immunotherapy and dacarbazine failed, she was enrolled in this study, and her primary resected tissues were processed for sequencing. This study identified *MAPRE2-RAF1* fusion with exon 5 of *MAPRE2* fused to exon 10 of *RAF1* (Figure 4), and she was administered vemurafenib for 1 month. Unfortunately, she showed progressive disease during vemurafenib administration.

## 4. Discussion

Our study revealed that 21.2% (25/118) of patients with gastrointestinal/rare cancers harbored at least one strong fusion by using a targeted RNA sequencing panel. In terms of gastrointestinal cancers including only CRC, GC, BTC, and pancreatic cancer, we found that 20.2% (18/89) of patients harbored at least one fusion gene. Notably, we identified 5.9% (7/118) patients with known targetable fusion genes involving *NTRK1* (*n*=3), *FGFR* (*n*=3), and *RET* (*n*=1) and 10.2% (12/118) of patients with potentially targetable fusion genes involving *RAF1* (*n*=4), *BRAF* (*n*=2), *ALK* (*n*=2), *ROS1* (*n*=1), *EGFR* (*n*=1), and *CLDN18* (*n*=2).

The first *NTRK1-TPM3* fusion was identified in colon cancer, and *NTRK* fusions have been identified in approximately 1% of all solid cancers across diverse cancer types [12, 13]. *NTRK* fusions are oncogenic drivers regardless of the tissue of origin, and first-generation *TRK* tyrosine kinase inhibitors (larotrectinib, entrectinib, or ropotrectinib) have demonstrated very promising antitumor efficacies in both adult and pediatric patients with *NTRK* fusion-positive cancers [13–15]. Larotrectinib induced a 75% response rate in *TRK* fusion-positive cancers, regardless of the tumor type, and was recently approved by the U.S. Food and Drug Administration for solid tumors with *NTRK* gene fusions [6]. We successfully identified 3 *NTRK* fusion-positive patients. Subsequently, one patient with angiosarcoma (no. 3) harboring *PEAR1-NTRK1* fusion was enrolled in the clinical trial of TRK inhibitor. Recently, the TRIDENT-1 trial demonstrated 8 confirmed cases of partial remission in TKI-naïve or TKI-pretreated *ROS1 *+* /NTRK* + patients at various dose levels [16].

*FGFR* fusions with multiple partners have been described in numerous cancer types. After the first report of *FGFR3-TACC3* fusion in glioblastoma, these fusions were reported in numerous solid cancers, including urothelial carcinoma, non-small-cell lung cancer, thyroid cancer, and uterine cervical carcinoma. Notably, *FGFR2* fusions are also present in 13–17% of intrahepatic cholangiocarcinomas [17–19]. A phase I trial of erdafitinib, an oral pan-fibroblast growth factor receptor (FGFR) inhibitor, demonstrated that urothelial carcinoma and cholangiocellular carcinoma were most responsive to erdafitinib showing objective response rates of 46.2% (12/26) and 27.3% (3/11), respectively, in patients with *FGFR* mutations and fusions [20]. Other *FGFR* inhibitors, such as BGJ398, showed an objective response rate of 14.8% (18.8% *FGFR2* fusions only) and disease control rate of 75.4% (83.3% *FGFR2* fusions only) in patients with *FGFR*-altered advanced cholangiocarcinoma [21]. This study successfully identified 3 patients with *FGFR* fusions (2 patients with *FGFR3-TACC3* fusion (nos. 4 and 5) and 1 patient with *FGFR2-NRAP* fusion (no. 16)); importantly, 2 of 3 patients harboring *FGFR* fusion had BTC, which may be responsive to erdafitinib or BGJ398 according to recent reports [20, 21].

*RET* fusions have been described in up to one-third of papillary thyroid cancers and in 2% of lung adenocarcinoma cases; *CCDC6-RET* and *NCOA4-RET* are the most commonly identified *RET* fusions [22, 23]. In CRC, Le Rolle et al. reported six *RET* fusion kinases among 3,117 advanced cases (0.2%) through comprehensive genomic profiling and identified *NCOA4-RET* fusion, which was consistent with the result for the patient with CRC in this study (no. 6) [24]. A recent study comparing *RET* fusion-positive and *RET* fusion-negative CRCs revealed that right-sided and MSI-high tumors are more likely to have *RET* fusion, and *RET* fusion is an independent poor prognostic factor in overall survival [25]. Confirmed responses to multikinase inhibitors with activity against *RET*, such as cabozantinib and vandetanib, can be achieved in some patients with lung cancer harboring *RET*-rearrangement or *RET*-mutation [26, 27]. Studies of RET-specific inhibitors such as BLU-667 have begun to show promising responses in early-phase clinical trials [6, 28].

*RAF* kinase fusions, such as of *BRA*F or *RAF1* (also known as *CRAF*), have been reported in various tumor types, including prostate cancer, GC, melanoma, and papillary thyroid cancer [1, 29]. *RAF* fusions activate the mitogen-activated protein kinase pathway, and a few reports have demonstrated the anticancer efficacy of MEK inhibitors in *RAF* fusion-positive melanoma [30, 31]. However, a recent report showed that existing *RAF* inhibitors cannot suppress *RAF1*-fusion-driven signaling pathways, and our study also showed that a melanoma patient harboring *MAPRE2-RAF1* fusion did not respond to vemurafenib [32]. Novel approaches to RAF1-directed targeted therapy should be explored.

Fusion between *CLDN18*, a tight junction gene, and *ARHGAP26*, a gene encoding an *RHOA* inhibitor, was first reported by the Cancer Genome Atlas to be enriched in the genomically stable subtype of GC [33]. Yao et al. detected *CLDN18-ARHGAP26* fusion in 3% of Asian GCs, and cancer cells transfected with this fusion showed reduced cell-cell adhesion and augmented invasiveness [11]. Recently, a Chinese group reported the largest dataset to date regarding signet ring cell carcinoma of GC; 17% of all signet ring cell carcinoma cases harbored this fusion gene and showed resistance to chemotherapy and worse survival outcomes [10]. Based on these results, *CLDN18-ARHGAP26* fusion is considered as a driver that contributes to aggressive tumor behavior and is a strong candidate for targeted drugs.

Our results suggest that detection of fusion genes using a targeted RNA sequencing panel can be beneficial for various cancer subtypes, particularly CRC and BTC. In patients with CRC, gene fusion is rarely observed, but recent studies showed that a substantial proportion of patients with CRC had potentially actionable gene rearrangements involving *ALK*, *ROS1*, and *NTRK* [9, 34]. Interestingly, this study also showed that 6 of 28 patients with CRC (21.4%) had targetable or potentially targetable gene rearrangements such as *NTRK1* (*n*=2), *RET* (*n*=1), *ALK* (*n*=1), *ROS1* (*n*=1), and *EGFR* (*n*=1) fusions. In patients with BTC, there are several ongoing clinical trials targeting *FGFR* (NCT03230318, NCT02052778, NCT01948297, NCT02924376, NCT02265341), *BRAF/MET* (NCT02034110), and *ALK/ROS1* (NCT02374489, NCT02034981, and NCT02568267). In this study, we found that 4 of 27 patients with BTC (14.8%) had targetable or potentially targetable gene rearrangements such as *FGFR3* (*n*=1), *FGFR2* (*n*=1), *BRAF* (*n*=1), and *ALK* (*n*=1) fusions. Unfortunately, patients with CRC and BTC harboring targetable or potentially targetable fusion genes in this study could not be administered targeted therapy because they were ineligible or respective clinical trials in Korea were not available.

## 5. Conclusions

In conclusion, we successfully identified a substantial proportion of patients harboring targetable (5.9%) and potentially targetable (10.2%) fusion genes by RNA panel sequencing in gastrointestinal and rare cancers. Involved fusion genes were *NTRK1*, *RET*, *FGFR3*, *FGFR2*, *BRAF*, *RAF1*, *ALK*, *ROS1*, and *CLDN18*. We suggest that detection of fusion genes by RNA panel sequencing can be beneficial in refractory patients with gastrointestinal or rare cancers, particularly in those with CRC and BTC.

## Figures and Tables

**Table 1:** Patient characteristics.

Variables	Total *N*=122	
	No	%
Sex		
Male	68	55.7
Female	54	44.3
Age, years		
Median (range), years	58 (31–81)	
Primary cancer site and histology		
Colorectal cancer (ADC)	28	23.0
Biliary tract cancer (ADC)	27	22.1
Gastric cancer (ADC)	25	20.5
Soft tissue sarcoma	18	14.8
Pancreatic cancer	9	7.4
GY cancer (ADC)	9	7.4
Ovarian cancer	6	4.9
Uterine cervical cancer	1	0.8
Fallopian tube cancer	1	0.8
Skin cancer	3	2.5
Melanoma	1	0.8
Skin squamous cell carcinoma	1	0.8
Trichilemmal carcinoma	1	0.8
Adenoid cystic carcinoma	1	0.8
Hepatocellular carcinoma	1	0.8
Pseudomyxoma peritonei	1	0.8
Urachal cancer	1	0.8
Initial stage		
Locoregional disease	67	54.9
Metastatic	55	45.1
Number of prior systemic treatment regimens		
1	32	26.2
2	31	25.4
3	24	19.7
≥4	26	21.3
Site of distant metastasis		
Liver	38	31.4
Peritoneal seeding	33	27.3
Lung	27	22.3
Lymph node	27	22.3
Bone	13	10.7
Ovary	9	7.4
Pleura	7	5.8

**Table 2:** Individual patients' information and concomitant genomic alterations.

No	Sex	Age	Cancer type	Histology	Strong fusion	SNV/indel
1	M	48	CRC	M/D ADC	TPM3 ⟶ NTRK1	KRAS G12V
2	F	74	CRC	M/D ADC	TPM3 ⟶ NTRK1,	
3	F	78	STS	Angiosarcoma	PEAR1 ⟶ NTRK1	
4	M	68	BTC	M/D ADC	FGFR3 ⟶ TACC3,	
5	M	58	GC	M/D ADC	FGFR3 ⟶ TACC3,	
6	F	53	CRC	M/D ADC	NCOA4 ⟶ RET	
7	F	32	GC	P/D ADC	INTERGENIC ⟶ CSMD1 ⟶ RAF1	
8	F	35	Melanoma	Skin melanoma	MAPRE2 ⟶ RAF1	
9	M	58	STS	Retroperitoneal leiomyosarcoma	IGH-AS ⟶ RAF1, AXL ⟶ LOC440300	
10	F	60	Pancreas cancer	M/D ADC	IQSEC1 ⟶ RAF1	KRAS G12D
11	M	56	CRC	SRCC	MAP7D1 ⟶ EGFR	KRAS G13D PIK3CA H1047R
12	M	57	CRC	W/D ADC	LACE1 ⟶ ROS1	KRAS G12A
13	M	80	BTC	P/D ADC	SP6 ⟶ ALK, THADA ⟶ VPS36	
14	M	65	CRC	W/D ADC	AXL ⟶ ALK	
15	M	66	GC	P/D ADC	AXL ⟶ IGH-AS	
16	M	56	BTC	M/D ADC	FGFR2 ⟶ NRAP	
17	M	56	GC	SRCC	CLDN18 ⟶ ARHGAP26	
18	M	47	GC	P/D ADC	CLDN18 ⟶ ARHGAP26	
19	M	67	BTC	ADC	BRAF ⟶ INTERGENIC ⟶ PTMA	
20	M	51	STS	Extraskeletal myxoid chondrosarcoma	EWSR1 ⟶ NR4A3, BRAF ⟶ UNALIGNED ⟶ LOC100996643	
21	M	50	STS	Liver leiomyosarcoma	CTBP2 ⟶ NOTCH2	
22	F	49	STS	Uterine leiomyosarcoma	ENO2 ⟶ ETV4	
23	M	56	STS	Retroperitoneal Malignant SFT	GNA13 ⟶ PRKCA	
24	F	61	BTC	P/D ADC	LOC100506217 ⟶ RELA	
25	M	52	CRC	M/D ADC	ESR1 ⟶ KIAA1731	

CRC: colorectal cancer; GC: gastric cancer; BTC: biliary tract cancer; STS: soft tissue sarcoma; W/D ADC: well-differentiated adenocarcinoma; M/D ADC: moderately differentiated adenocarcinoma; P/D ADC: poorly differentiated adenocarcinoma; SRCC: signet ring cell carcinoma.

## Data Availability

The clinicopathological data used to support the findings of this study are available from the corresponding author upon request.

## Supplementary Materials

Supplementary Materials Supplementary Table 1: the list of the 36 target genes of Comprehensive Thyroid & Lung kit. Supplementary Table 2: the list of the 53 target genes of Solid Tumor kit.
