# Supplementary material for: Detection of Fusion Genes Using a Targeted RNA Sequencing Panel in Gastrointestinal and Rare Cancers
Source: J Oncol. 2020 Jan 22;2020:4659062. doi: 10.1155/2020/4659062 (PMC7204148; doi:10.1155/2020/4659062)
Supplement: Supplementary Materials — Supplementary Table 1: the list of the 36 target genes of Comprehensive Thyroid & Lung kit. Supplementary Table 2: the list of the 53 target genes of Solid Tumor kit. [file 4659062.f1.docx]

**Supplementary Table 1.** The list of the 36 target genes of Comprehensive Thyroid & Lung kit

| AKT1 | FGFR1 | MAP2K1 | RET |
| --- | --- | --- | --- |
| ALK | FGFR1 | MET | ROS1 |
| AXL | FGFR3 | NRAS | SLC5A5 |
| BRAF | GNAS | NRG1 | THADA |
| CALCA | HRAS | NTRK1 | TTF1 |
| CCND1 | IDH1 | NTRK2 |  |
| CTNNB1 | IDH2 | PIK3CA |  |
| DDR2 | KRAS | PPARG |  |
| EGFR | KRT20 | PTH |  |
| EERBB2 | KRT7 | RAF1 |  |

https://archerdx.com/comprehensive-thyroid-and-lung-ctl/

**Supplementary Table 2.** The list of the 53 target genes of Solid Tumor kit

| AKT3 | ETV1 | MAML2 | NTRK1 | PRKCA | TFEB |
| --- | --- | --- | --- | --- | --- |
| ALK | ETV4 | MAST1 | NTRK2 | PRKCB | THADA |
| ARHGAP26 | ETV5 | MAST2 | NTRK3 | RAF1 | TMPRSS2 |
| AXL | ETV6 | MET | NUMBL | RELA |  |
| BRAF | EWSR1 | MSMB | NUTM1 | RET |  |
| BRD3 | FGFR1 | MUSK | PDGFRA | ROS1 |  |
| BRD4 | FGFR2 | MYB | PDGFRB | RSPO2 |  |
| EGFR | FGFR3 | NOTCH1 | PIK3CA | RSPO3 |  |
| ERG | FGR | NOTCH2 | PKN1 | TERT |  |
| ESR1 | INSR | NRG1 | PPARG | TFE3 |  |

<https://archerdx.com/solid-tumor-2/>
